# Oral vinorelbine and capecitabine as first-line therapy in metastatic breast cancer: a retrospective analysis

**DOI:** 10.2144/fsoa-2020-0095

**Published:** 2021-11-12

**Authors:** Maria Rosaria Valerio, Pietro Spadaro, Concetta Arcanà, Nicolò Borsellino, Calogero Cipolla, Paolo Vigneri, Dario Piazza, Vittorio Gebbia

**Affiliations:** 1 Medical Oncology Unit, Policlinico Paolo Giaccone, University of Palermo, Italy; 2 Medical Oncology Unit, Casa di Cura Villa Salus, Messina, Italy; 3 Medical Oncology Unit, Hospital Buccheri La Ferla, Palermo, Italy; 4 Surgical Oncology Unit, Policlinico Paolo Giaccone, Palermo, Italy; 5 Medical Oncology Unit, Policlinico, Catania, Italy; 6 GSTU Foundation for the Study of Tumors, Palermo, Italy; 7 Department Promise, University of Palermo, Palermo, Italy; 8 Medical Oncology Unit, La Maddalena Clinic for Cancer, Palermo, Italy

**Keywords:** breast carcinoma, capecitabine, metastases, oral chemotherapy, vinorelbine

## Abstract

A retrospective analysis of 70 patients with triple-negative or hormone-resistant advanced breast carcinoma who had not previously received chemotherapy was carried out. Patients received oral vinorelbine 60 mg/m^2^ on day 1 and 8, plus capecitabine 1000 mg/m^2^ bid for 14 consecutive days every 3 weeks. Overall response rate was 53% with a 9% complete response rate. Stable disease was recorded in 27% of the cases. Median progression-free survival was 7.9 months and median overall survival was 29.2 months. Toxicity was generally mild and easily manageable. These data demonstrate that this combination is feasible, safe and active as first-line treatment of triple-negative fully hormone-resistant advanced breast carcinoma patients.

## Lay abstract

The adoption of metronomic chemotherapy in the treatment of breast cancer is an important step forward in the management of this disease. The fluoropyrimidine capecitabine and the vinca alkaloid vinorelbine have been shown to be at least as active. Our data confirm the activity and safety of this all-oral regimen as first-line metronomic therapy being response rate.

Intravenous administration of chemotherapy (CT) represents a significant physical, emotional and social burden for both patients and health providers in terms of complications and economic costs associated with the implant and management of indwelling venous access catheters and pumps as well as the growing numbers of hospital accesses for intravenous (iv.) therapy [1]. Several studies have clearly shown that patients themselves generally prefer oral CT if equal efficacy to iv. therapy is assured [1]. Therefore, all oral CT represents a very reasonable therapeutic option in consideration of the palliative nature of the treatment of metastatic breast cancer (MBC). The adoption of metronomic chemotherapy in the treatment of breast cancer is an important step forward in the management of this disease. Thus, the continuous administration of low-dose drugs allows to increase the time of treatment, minimizing the risk of side effects. Clinical experience and data from several studies suggest that this approach should be offered at the present time primarily to patients with HR positive, while waiting for data from ongoing studies on triple-negative and HER2-positive disease [2].

The fluoropyrimidine capecitabine (CAP) and the vinca alkaloid vinorelbine (VNR) have been shown to be at least as active as iv. counterparts. The two drugs have been compared in an European Organisation for Research and Treatment of Cancer (EORTC) prospective Phase II trials in patients pretreated with taxanes and anthacyclines showing equi-activity but a different toxicity profile, being hand foot syndrome and diarrhea more frequent with CAP and neutropenia more incident for VNR [3]. Dose-finding studies of oral VNR plus CAP have consistently shown that the combination is feasible and well tolerated. Severe neutropenia was the dose-limiting toxicity and the recommended doses of VNR and CAP were respectively 80 mg/m^2^ day 1 and 8 and 1000–1200 mg/m^2^ bid day 1→14 every 3 weeks [4].

This all oral regimen has been tested with good results [5], but in this paper we report our experience in a challenging setting such as first-line treatment of triple-negative or fully hormone-resistant HER-negative MBC.

## Materials & methods

### Study design

Patients with triple-negative or hormone-refractory MBC treated with oral VNR/CAP as first-line treatment were anonymously collected and retrospectively analyzed for clinical efficacy and toxicity after communication to the ethical committees from January 2008 up to December 2014. Patients lacking clinical and/or radiological evidence of response and/or certain data for time-related parameters were excluded from final analysis. Clinical data for all patients were submitted for external review.

### Eligibility criteria

Eligible patients included in this retrospective analysis had to meet the following entry criteria: age ≥18 years, performance status ≤according to the ECOG scale, pathologically confirmed diagnosis of MBC, no previous treatment with chemotherapy for advanced and/or metastatic disease while previous adjuvant chemotherapy with anthracyclines and/or taxanes were allowed. Patients with positive estrogen receptors could have received multiple lines of hormonotherapy. Blood cell counts had to be permissive for chemotherapy (white blood cells [WBCs] >3500/mmc; platelet [PLT] >100,000/mmc). Renal (blood urea nitrogen [BUN] <50 mg %; serum creatinine <1,2 mg %) and liver (serum bilirubin <1.2 mg %; serum transaminases within two-times the normal values) functions had to be within the normal limits. Metastatic disease had to be also measurable disease according to the RECIST criteria [4] with the absence of clinically detectable deposits in the CNS. Patients were excluded if osteoblastic bone lesion or ascites were the only sites of disease. No history of previous malignancies other than basal cell skin cancer or curatively treated carcinoma *in situ* of the cervix was allowed as well as severe and uncontrolled metabolic, infectious, cardiological or neurological disease.

### Chemotherapy schedules

Patients received oral VNR 60 mg/m^2^ on day 1 and 8 with water 30 min after a meal plus CAP 1000 mg/m^2^ twice daily for 14 consecutive days followed by 1-week rest. Oral anti-HT3 drugs were employed before VNR administration, while no prophylaxis for emesis was employed for CAP administration. In case of nausea and/or vomiting related to CAP patients were treated with oral metoclopramide. Both patients and caregivers were informed to report any side effect weekly, according to our institution procedures for oral antineoplastic treatments. Data of hematological toxicity or other serum chemistry test were inferred from analysis routinely done before every CT administration.

### Patients evaluation

Patients were staged for disease extension and response evaluation with physical examination, chest x-rays, chest and abdominal CT scan, sonograms, bone scans, complete blood counts and serum chemistry tests as needed. Objective responses were recorded according to the RECIST criteria and results were reported as best overall response [6]. The sum of complete and partial responses (CR and PR) was defined as the overall response rate (ORR). The sum of ORR and stabilization of disease (SD) was defined as the tumor growth control rate (TGCR).

### Safety evaluation

Evaluation of tolerance and side effects was carried out according to National Cancer Institute Common Toxicity Criteria (NCI CTC) version 2.0 criteria by clinical and laboratory investigations. Dose modifications were performed in a very flexible way depending on the type, severity and duration of side effects. Patients and caregivers were required to report toxicity employing a dedicated fax or telephone line. Complete blood counts were obtained before any oral VNR administration. CAP was stopped in case of grade 3–4 skin of gastrointestinal toxicity and subsequently administered with a 25–50% dosage reduction. Oral VNR was reduced by 25% if grade-4 hematological toxicity. Granulocyte colony-stimulating factor (G-CSF) was employed according to clinical needs and physician's decision.

### Statistical methods

Response duration was calculated from the start of CT until the date when progression was evidenced, or last follow-up evaluation or death. Progression-free survival (PFS) was calculated from the date of first CT cycle until progression. Overall survival (OS) was calculated from the first day of CT to the time of death or last follow-up evaluation. Objective response was evaluated as relative rates with their 95% confidence limits (95% CL). A univariate analysis of survival data according to product-limit estimate (Kaplan–Meier) was performed employing the computer statistical software Prism (Graph Pad Incorporated, CA, USA). Calculation of dose intensity was carried out according to Hryniuk [7].

## Results

### Patient population

Between January 2008 and December 2014, 94 patients were treated in four centers. However, 70 patients (80%) were considered evaluable and 24 were excluded from the analysis because of incomplete objective response and survival data.

Baseline characteristics of the 70 evaluable patients are shown in Table 1. All patients had recurrent disease after more than 12 months after the completion of an adjuvant therapy. Thirty-seven patients (54%) had dominant visceral metastatic disease and 33 (47%) patients had extra-visceral dominant disease. Thirty patients (43%) had triple-negative metastatic disease. Forty patients (57%) with hormone-refractory MBC had previously received first-line hormonotherapy with an aromatase inhibitor but one patient who received tamoxifen, and a second-line treatment with high-dose fulvestrant. Among these patients with hormone-positive MBC 18 cases (26%) received a third-line hormonotherapy with everolimus plus exemestane. Lack of response to hormonal manipulations accordingly to treating physician decision classified patients as hormone-refractory.

**Table 1.** Patient baseline characteristics.

Patients clinical and demographic characteristics	Patients (n)	Percent
Enrolled patients	70	100
Median age, years (range)	63 (46–76)	
Performance status: ECOG 0 ECOG 1 ECOG 2	48 14 8	69 21 12%
Histology: Ductal infiltrating carcinoma Lobular carcinoma	66 4	94 6
Hormone receptors: Positive Triple negative	40 30	57 43
HER status negative^†^	70	100
Previous treatments: Surgery Radiotherapy Adjuvant hormonal therapy Adjuvant chemotherapy Hormonal therapy (advanced disease) Aromatase inhibitors Tamoxifen Faslodex Everolimus - examestane	68 61 40 61 40 39 1 40 18	97 87 57 87 57 56 1 57 26
Type of chemotherapy: Anthracyclines axanes CMF	18 43 6	26 61 9
Sites of disease: Bone Lung Liver Nodes Skin	41 16 21 24 5	59 23 30 34 7
Metastatic sites (n): 1 2 ≥3	15 30 25	21 43 36

† Other than HER 3+.

CMF: Cyclophosphamide, methotrexate, 5-fluorouracil; ECOG: Eastern Cooperative Oncology Group.

### Clinical efficacy

**Figure 1.**
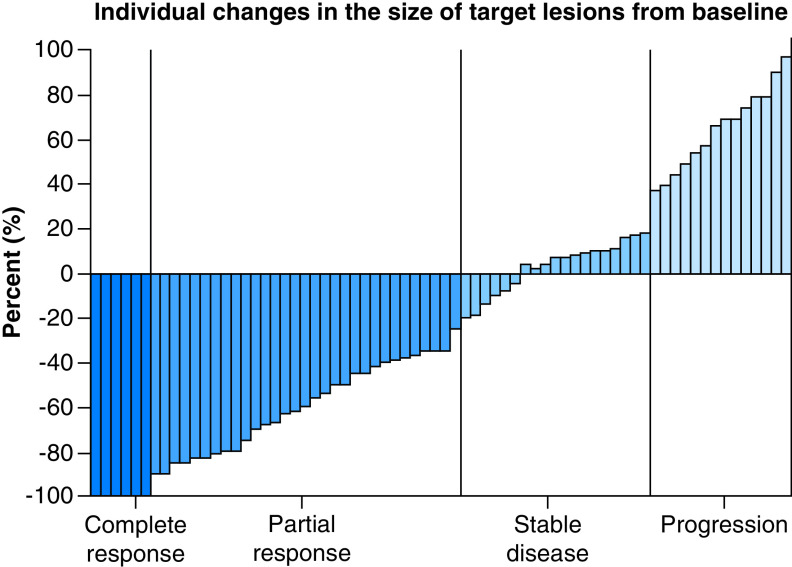
Tumor shrinkage.

Six out of 70 patients fully evaluable for response efficacy achieved a CR (9%; 95% CL: 0–31.0), 31 patients showed a PR (44%; 95% CL: 30.9–57.1), for an ORR of 53% (95% CL: 41.9–64.1) (Table 2). Nineteen patients were categorized as SD (27%; 95% CL: 9.9–44.1) and 14 patients as PD (20%; 95% CL: 1.2–38.8). TGCR was 80% (95% CL: 75.3–84.7). Deepness of response is shown in Figure 1. Median PFS was 7.9 months (range: 2–15 months) and median OS was 29.2 months (range: 13–41 months). No statistically significant relationship was found between site or number of metastases and objective response, or between triple-negative and hormone-resistant patients. ORR was 50% in the group of TNBC and 55% in the hormone-refractory one.

**Table 2.** Treatment outcomes.

Objective response (RECIST criteria)			
	Patients (n)	Percent	95% CL
Evaluable patients	70	100%	
Overall response	37	53%	41.9–64.1
Complete response	6	9%	0–31.0
Partial response	31	44%	30.9–57.1
Stable disease	19	27%	9.9–44.1
Tumor growth control	56	80%	75.3–84.7
Progressive disease	14	20%	1.2–38.8

**Survival parameters**			
	Median	Range	
Progression-free survival	7.9 months	2–15 months	
Overall survival	29.2 months	13–41 months	

CL: Confidence limits.

### Safety

Side effects are depicted in Table 3. Globally 411 cycles were administered with a median of 5.9 cycle/patient (range: 3–12). No chemotherapy-related deaths as well as treatment discontinuations due to toxicity were observed. Of the 70 patients analyzed, 19% withdrew from treatment after the third cycle because of the occurrence of progressive disease. Grade 3 and 4 leukopenia and neutropenia were recorded in 21 and 16% of cases respectively, but G-CSF administration was required only in four cases. Overall, toxicity-related dose reductions were made in 16 patients mainly due to white blood cell and/or platelet toxicity or hand-foot syndrome (HFS). Median duration of interval among courses was 22.6 days. The planned dose-intensity was 40 mg/m^2^/week for oral VNR and 9333 mg/m^2^/week for CAP. The received, relative median dose-intensity of oral VNR and CAP were 0.94 (37.6 mg/m^2^/week) and 0.89 (8306 mg/m^2^/week), respectively. Statistical analysis performed in the attempt to correlate response rate with dose-intensity was not significant.

**Table 3.** Toxicity according to National Cancer Institute Common Toxicity Criteria.

Toxicities	Grade 1		Grade 2		Grade 3		Grade 4	
	Patients (n)	Percent	Patients (n)	Percent	Patients (n)	Percent	Patients (n)	Percent
Leukopenia	29	41%	9	13%	11	16%	4	6%
Neutropenia	15	21%	9	13%	8	11%	3	4%
Anemia	16	23%	---	---	---	---	---	---
Platelets	13	19%	6	9%	3	4%	---	---
Nausea/vomiting	23	33%	7	10%	---	---	---	---
Mucositis	6	9%	2	3%	---	---	---	---
Transaminases	6	9%	2	3%	---	---	---	---
Peripheral neuropathy	10	14%	---	---	---	---	---	---
Abdominal pain	7	10%	1	1%	---	---	---	---
Diarrhea	15	21%	6	9%	---	---	---	---
Hand foot syndrome	12	17%	5	7%	2	3%	---	---

## Discussion

In this article we report our experience with an all-oral regimen of VNR 60 mg/m^2^ on days 1 and 8 plus CAP 1000 mg/m^2^ bid for 14 consecutive days every 3 weeks in a series of 70 patients with triple-negative or hormone-resistant HER-2 negative MBC previously untreated with CT for advanced disease. In our hands, this all-oral combination resulted active and very well tolerated. As shown in Table 2, overall a CR was recorded in 9% of patients, PR in 44% and SD in 27% of cases, for a TGCR of 80%. Median PFS and overall survival were 7.9 and 29.2 months respectively. This multicentric study, albeit retrospective, demonstrates the activity of this combination in a challenging clinical setting such as triple-negative MBC patients and in hormone-resistant patients progressing after several lines of hormonal manipulations. This report is interesting since data in triple-negative patients with such all-oral chemotherapy are scarce in medical literature although there is a recent scientific leap oriented to fill this gap, especially in the perspective of precision medicine [8–10]. Moreover published trials often include very low numbers of triple-negative patients with the exception of the paper of Campone *et al.* [11].

Overall, our data confirm the activity and safety of this all-oral regimen as first-line metronomic therapy being response rate, survival parameters and toxicity in the range reported in medical literature by other authors [11–19]. Table 4 shows the main studies reporting the activity and toxicity of this all-oral combination of VNR and CAP. All studies, but one, consistently reported an ORR ≥51% (range: 51–76%). Only the study by Gampenrieder *et al.* reported a somewhat lower ORR of 36.7% but authors employed a dose of CAP of only 500 mg/m^2^ bid which may explain the lower activity [15]. However other authors have shown that ORR with oral VNR + CAP is not statistically different between patients who received more or less than the median dose intensity, with no difference in OS or PFS. The use of lower doses than those currently recommended – if clinically advisable – should be not detrimental in terms of efficacy. In 2016 Cazzaniga *et al.* reported an open label Phase II study on the all-oral combination of VNR and CAP on a true metronomic schedule reporting a quite low rate of grade 3–4 toxicity per cycle, being non febrile neutropenia and hand-foot syndrome the most frequently reported side effects. Out of 35 patients treated in first-line only 13 had TNBC and their outcome in terms of time-to-progression was very similar to those with hormone sensitive disease. The status of hormone refractoriness and the number of previous lines was of hormonal manipulation were unclear. However, these results are superimposable to those achieved in the present experience.

**Table 4.** Studies reporting the activity and toxicity of first-line all-oral combination of vinca alkaloid vinorelbine and capecitabine.

Study (year)	Patients (n)	Adjuvant chemotherapy	Schedule	ORR	Median PFS (months)	Median OS (months)	Grade 3–4 toxicity (>10%)	Ref.
Nolè *et al.* (2009)	42	78.8% anthracyclines 60.5% Taxanes 5.8%	VNR 60 mg/m^2^ Day 1 + 8 + 15 q21 days CAP 1 gr/m^2^ bid Day 1→14	54.8%	8.4	25.8	Neutropenia G3 21%; G4 25%	[13]
Tubiana-mattieu *et al.* (2009)	49	63% anthracyclines 37.1% Taxanes 5.8%	VNR 60–80 mg/m^2^ Day 1 + 8 q21 days CAP 1 gr/m^2^ bid Day 1→14 750 mg/m^2^ >65 years	51.0%	8.4	29.2	Neutropenia G3 26%; G4 23%	[14]
Finek *et al.* (2009)	58	Anthracyclines (mostly)	VNR 60 mg/m^2^ Day 1 + 8 q21 days CAP 1 gr/m^2^ bid Day 1→14	56.5%	10.5	17.5	-	[12]
Gampenrieder *et al.* (2010)	24	59.4% anthracyclines 53% Taxanes 34%	VNR 60 mg/m^2^ Day 1 + 8 q21 days CAP 500 mg/m^2^ bid Day 1→14	36.7%	8.0 TTP	32.0	Neutropenia G3 12%	[15]
Hassan *et al.* (2010)	31	Anthracyclines 100%	VNR 60 mg/m^2^ Day 1 + 8 q21 days CAP 1 gr/m^2^ bid Day 1→14	67%	7.8	21	Neutropenia G3–4 16% Diarrhea G3–4 12.6% HFS G3–4 22.4%	[16]
Strada *et al.* (2012)	46	97.8% anthracyclines 30.4% Taxanes 65.2%	VNR 60 mg/m^2^ Day 1 + 8 q21 days CAP 1 gr/m^2^ bid Day 1→14	76.0%	8.4	34.3	Neutropenia G3 13% Leukopenia G3 10.9%	[17]
Tawfik *et al.* (2013)	28	anthracyclines 100% Taxanes 60%	VNR 60 mg/m^2^ Day 1 + 8 q21 days CAP 1 gr/m^2^ bid Day 1→14	57.1%	8.6	27.2	Neutropenia G3 21.4% Vomiting G3 10.7%	[18]
Campone *et al.* (2013)	44	anthracyclines 100% Taxanes 18.2%	VNR 60 mg/m^2^ Day 1 + 8 q21 days CAP 1 gr/m^2^ bid Day 1→14	31.8%	7.2	22.2	Neutropenia G3–4 47.7% Leukopenia G3–4 29.6%	[11]
Cazzaniga *et al.* (2016)	35	Not specified	VNR 40 mg fixed dose three-times/week CAP 15007 day continuously	35.5%	7.9 TTP	Not reported	Neutropenia 1.6% of cycles Hand foot syndrome 1% of cycles	[14]
Cinieri *et al.* (2017)	49	Anthracyclines 80%	VNR 60 mg/m^2^ Day 1 + 8 q21 days CAP 1 gr/m^2^ bid Day 1→14	67.3%	7.6	30.2	Neutropenia G3 25%; G4 25% Febrile Neutropenia G4 12% Fatigue G3 12% Vomiting G3 10%	[15]
Present study	52	90% anthracyclines 21% Taxanes 71%	VNR 60 mg/m^2^ Day 1 + 8 q21 days CAP 1 gr/m^2^ bid Day 1→14	58%	7.8	29.5	Neutropenia G3 15% Leukopenia G3 12%	

Reported data refer only to evaluable patients.

ORR: Overall response rate; OS: Overall survival; PFS: Progression-free survival; TTP: Time to progression.

As shown in Table 3 both pattern and severity of side effects reported in our experience fit in the range reported by other authors. Hematological and gastrointestinal toxicity were the most common side effects and, in most cases, easily manageable. Severe grade 3–4 toxicities were only occasionally reported in a minority of cases. The good tolerability of the regimen is further strengthened by data reported by Rousseau *et al.*, which tested the combination in a series of 80 patients older than 70 years with advanced cancer of the breast, lung and prostate [20]. In this series the functional status measured by activities of daily living was stabilized or improved in 82% of patients after three cycles of CT with excellent compliance in 69% of cases.

## Conclusion

**Figure 2.**
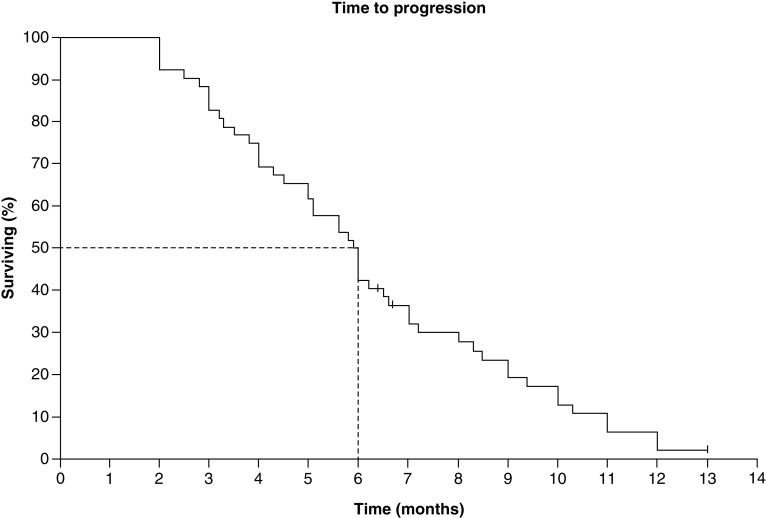
Time to Progression.

Despite recent therapeutic progress, systemic chemotherapy still maintains a pivotal role in the management of advanced TNBC. Scientific advances have recently provided a sound rationale for other treatment approaches for TNBC, such as the use of immunotherapy and poly(ADP-ribose) polymerase inhibitors. These two classes of drugs have shown promising results but have yet to demonstrate a proven OS benefit. Hopefully, other agents, such as antibody-drug conjugates and targeted therapies, will represent the next frontier in the treatment of this disease [21–24] (Figure 2).

## Future perspective

In conclusion the above-reported data suggest that oral metronomic VNR plus CAP may be useful in the palliative treatment of patients with triple-negative and hormone-resistant MBC especially in order to reducing patients toxicity burden as may happen in elderly patients or in those with expect poor tolerance to taxanes with or without biologics. Another potential advantage of this metronomic approach may be represented by reduction of the affluence to the outpatient infusions clinic and or reducing difficulties to patients with geographical accessibility. Further studies are; however, needed to optimize and possibly improve efficacy of oral treatment for MBC.

**Summary points**

- The adoption of metronomic chemotherapy in the treatment of breast cancer is an important step forward in the management of this disease.
- The fluoropyrimidine capecitabine and the vinca alkaloid vinorelbine have been shown to be at least as active.
- Our data confirm the activity and safety of this all-oral regimen as first-line metronomic therapy being response rate.
